# Intronic *FGF14* GAA repeat expansions are a common cause of ataxia syndromes with neuropathy and bilateral vestibulopathy

**DOI:** 10.1136/jnnp-2023-331490

**Published:** 2023-06-30

**Authors:** David Pellerin, Carlo Wilke, Andreas Traschütz, Sara Nagy, Riccardo Currò, Marie-Josée Dicaire, Hector Garcia-Moreno, Mathieu Anheim, Thomas Wirth, Jennifer Faber, Dagmar Timmann, Christel Depienne, Dan Rujescu, José Gazulla, Mary M Reilly, Paola Giunti, Bernard Brais, Henry Houlden, Ludger Schöls, Michael Strupp, Andrea Cortese, Matthis Synofzik

**Affiliations:** 1 Department of Neuromuscular Diseases, UCL Queen Square Institute of Neurology and The National Hospital for Neurology and Neurosurgery, University College London, London, UK; 2 Department of Neurology and Neurosurgery, Montreal Neurological Hospital and Institute, McGill University, Montreal, QC, Canada; 3 Research Division Translational Genomics of Neurodegenerative Diseases, Hertie-Institute for Clinical Brain Research and Center of Neurology, University of Tübingen, Tübingen, Germany; 4 German Center for Neurodegenerative Diseases (DZNE), Tübingen, Germany; 5 Department of Neurology, University Hospital Basel, University of Basel, Basel, Switzerland; 6 Department of Brain and Behavioral Sciences, University of Pavia, Pavia, Italy; 7 Ataxia Centre, UCL Queen Square Institute of Neurology, University College London, London, UK; 8 National Hospital for Neurology and Neurosurgery, University College London Hospitals NHS Foundation Trust, London, UK; 9 Service de Neurologie, Hôpitaux Universitaires de Strasbourg, Hôpital de Hautepierre, Strasbourg, France; 10 Fédération de Médecine Translationnelle de Strasbourg (FMTS), Université de Strasbourg, Strasbourg, France; 11 Department of Neurology, University Hospital Bonn, Bonn, Germany; 12 German Center for Neurodegenerative Diseases (DZNE), Bonn, Germany; 13 Department of Neurology and Center for Translational Neuro- and Behavioral Sciences (C-TNBS), Essen University Hospital, University of Duisburg-Essen, Essen, Germany; 14 Institute of Human Genetics, Essen University Hospital, University of Duisburg-Essen, Essen, Germany; 15 Department of Psychiatry and Psychotherapy, Comprehensive Center for Clinical Neurosciences and Mental Health, Medical University of Vienna, Vienna, Austria; 16 Department of Neurology, Hospital Universitario Miguel Servet, Zaragoza, Spain; 17 Department of Human Genetics, McGill University, Montreal, QC, Canada; 18 Centre de Réadaptation Lucie-Bruneau, Montreal, QC, Canada; 19 Department of Neurodegenerative Diseases, Hertie-Institute for Clinical Brain Research and Center of Neurology, University of Tübingen, Tübingen, Germany; 20 Department of Neurology and German Center for Vertigo and Balance Disorders, LMU University Hospital, LMU Munich, Munich, Germany

**Keywords:** CEREBELLAR ATAXIA, NEUROPATHY, NEUROGENETICS, VERTIGO, MOVEMENT DISORDERS

## Abstract

**Background:**

Intronic GAA repeat expansions in the fibroblast growth factor 14 gene (*FGF14*) have recently been identified as a common cause of ataxia with potential phenotypic overlap with *RFC1*-related cerebellar ataxia, neuropathy and vestibular areflexia syndrome (CANVAS). Our objective was to report on the frequency of intronic *FGF14* GAA repeat expansions in patients with an unexplained CANVAS-like phenotype.

**Methods:**

We recruited 45 patients negative for biallelic *RFC1* repeat expansions with a combination of cerebellar ataxia plus peripheral neuropathy and/or bilateral vestibulopathy (BVP), and genotyped the *FGF14* repeat locus. Phenotypic features of GAA-*FGF14*-positive versus GAA-*FGF14*-negative patients were compared.

**Results:**

Frequency of *FGF14* GAA repeat expansions was 38% (17/45) in the entire cohort, 38% (5/13) in the subgroup with cerebellar ataxia plus polyneuropathy, 43% (9/21) in the subgroup with cerebellar ataxia plus BVP and 27% (3/11) in patients with all three features. BVP was observed in 75% (12/16) of GAA-*FGF14*-positive patients. Polyneuropathy was at most mild and of mixed sensorimotor type in six of eight GAA-*FGF14*-positive patients. Family history of ataxia (59% vs 15%; p=0.007) was significantly more frequent and permanent cerebellar dysarthria (12% vs 54%; p=0.009) significantly less frequent in GAA-*FGF14*-positive than in GAA-*FGF14*-negative patients. Age at onset was inversely correlated to the size of the repeat expansion (Pearson’s r, −0.67; R^2^=0.45; p=0.0031).

**Conclusions:**

GAA-*FGF14*-related disease is a common cause of cerebellar ataxia with polyneuropathy and/or BVP, and should be included in the differential diagnosis of *RFC1* CANVAS and disease spectrum.

## Introduction

Dominantly inherited intronic GAA repeat expansions in the fibroblast growth factor 14 gene (*FGF14*) have recently been shown to be a common cause of hereditary ataxia (GAA-*FGF14*-related disease; spinocerebellar ataxia 27B (MIM: 620 174)).^1 2^ Initial observations of cerebellar ataxia and bilateral vestibulopathy (BVP) in a subset of patients carrying an *FGF14* GAA repeat expansion suggested partial phenotypic overlap between GAA-*FGF14*-related disease and cerebellar ataxia, neuropathy and vestibular areflexia syndrome (CANVAS).^1 2^ Biallelic intronic pentanucleotide repeat expansions in the replication factor C subunit 1 gene (*RFC1*) are a frequent cause of CANVAS, accounting for 70% to 100% of cases in various series.^3 4^ Phenotypic analysis of *RFC1*-positive patients has shown that CANVAS is not a strictly delineated disease entity but rather a phenotypic cluster occurring along a continuum of variable involvement of the cerebellar, sensory and vestibular systems.^5–8^ While biallelic *RFC1* repeat expansions are the main cause of CANVAS-spectrum disease, other causative genes are yet to be identified, especially in the subgroup of patients with partial features of CANVAS.^4 9^

Here, we studied the frequency of *FGF14* GAA repeat expansions in patients with a combination of cerebellar ataxia plus peripheral neuropathy and/or BVP negative for biallelic *RFC1* repeat expansions, and report on the phenotypic spectrum of GAA-*FGF14*-positive patients.

## Methods

### Patient enrollment

Forty-five index patients with neurodegenerative ataxia for which an underlying genetic cause had not yet been identified were recruited from seven different centres in Europe (France: 1, Germany: 4, Spain: 1, UK: 1 centre). To be eligible for inclusion in the study, patients needed to have cerebellar ataxia plus polyneuropathy confirmed by nerve conduction studies (excluding focal entrapment neuropathies) and/or BVP evidenced by reduced bilateral vestibulo-ocular reflex by bedside head impulse test or video head impulse test (vHIT); and negative results on screening for biallelic *RFC1* repeat expansions. The bedside head impulse test, performed by experienced neurologists with expertise in ataxia, was available in 38 of 45 (84%) patients, the vHIT was available in 21 of 45 (47%) patients and either test was available in 39 of 45 (87%) patients. Results of brain MRI and nerve conduction studies were available for review in 82% (37/45) and 80% (36/45) of patients, respectively. Deep phenotyping was performed through review of medical records and, when possible, patient re-evaluation using a standardised data sheet for both GAA-*FGF14*-positive and GAA-*FGF14*-negative patients.

### Genetic screening for *RFC1* and *FGF14* repeat expansions

Screening for *RFC1* repeat expansions was performed as described previously.^3^ The *FGF14* repeat locus was genotyped by long-range PCR. Repeat sizes were measured by capillary electrophoresis of fluorescent long-range PCR amplification products, as described previously.^10^ Results of fragment length analysis were confirmed by agarose gel electrophoresis of PCR amplification products. Patients who had large amplification products by PCR underwent bidirectional repeat-primed PCRs targeting the 5’-end and the 3’-end of the locus to ascertain the presence of a GAA repeat expansion.^10^ Expansions of at least 250 GAA repeat units were considered pathogenic.^1 2^

### Data availability

Individual deidentified patient data may be shared at the request of any qualified investigator on reasonable request.

## Results

**Figure 1:**
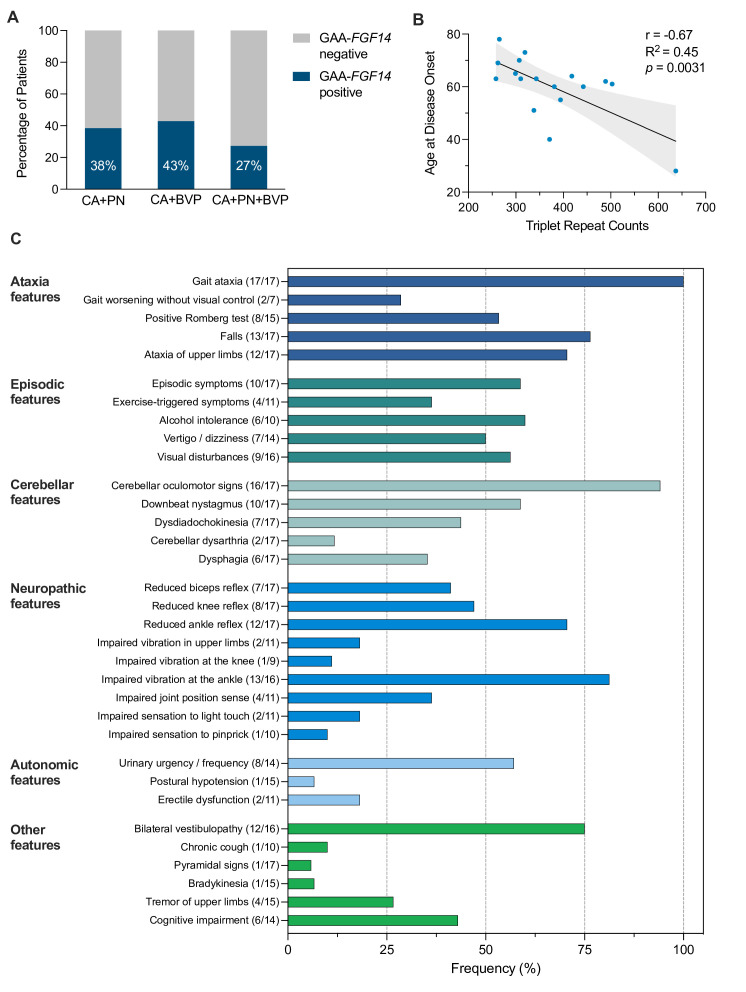
Frequency of the *FGF14* GAA repeat expansion, age at onset correlation and clinical features of GAA-*FGF14*-positive patients. (A) Percentage of patients who carried an *FGF14* (GAA)_≥250_ repeat expansion in the subgroups with (1) cerebellar ataxia plus polyneuropathy (CA+PN) (5 of 13 patients), (2) cerebellar ataxia plus bilateral vestibulopathy (CA+BVP) (9 of 21) and (3) cerebellar ataxia plus polyneuropathy and bilateral vestibulopathy (CA+PN+ BVP) (3 of 11). (B) Inverse correlation between size of the *FGF14* repeat expansion and age at disease onset in 17 patients (Pearson’s r, −0.67; R^2^=0.45; p=0.0031). The grey area displays the 95% CI. Simple linear regression fitting (slope, −0.079 and intercept, 89.84) suggests that age at onset decreases by about 3.96 years (95% CI: 1.56 to 6.37 years) for every increment of 50 GAA repeats above the pathogenic threshold of 250 repeat units. (C) Frequency of individual phenotypic features in 17 GAA-*FGF14*-positive patients. Numbers in brackets indicate the number of affected patients over the total number of patients assessed for this feature. *FGF14*, fibroblast growth factor 14 gene.

Of the 45 patients enrolled in this study, 17 (38%) carried a heterozygous *FGF14* (GAA)_≥250_ repeat expansion (median size of expansion, 343 repeat units; range, 258–637 repeat units). Repeat expansions were present in 38% of patients with cerebellar ataxia plus polyneuropathy (5/13), 43% of patients with cerebellar ataxia plus BVP (9/21) and 27% of patients with all three features (3/11) (figure 1A). While no patient met the proposed diagnostic criteria for clinically probable or definite CANVAS, 1 GAA-*FGF14*-positive and 2 GAA-*FGF14*-negative patients fulfilled the criteria for clinically possible CANVAS.^9^

Median age of onset was 63 years (range, 28–78 years) in the GAA-*FGF14*-positive cohort. We observed an inverse correlation between the age at onset and the size of the repeat expansion (17 patients; Pearson’s r, −0.67; R^2^=0.45; p=0.0031) (figure 1B). Clinical cerebellar features predominantly included gait ataxia (100%), cerebellar oculomotor signs (94%) and upper limb ataxia (71%) (figure 1C). Brain MRI of 10 patients showed cerebellar atrophy (10/13; 77%), which was limited to the vermis in 3 patients and extended to the hemispheres in 7 patients. The vHIT confirmed bilateral vestibular hypofunction in all eight patients in whom it was performed. Chronic cough was rarely observed in GAA-*FGF14*-positive patients (1/10; 10%). Of the eight patients with polyneuropathy confirmed by nerve conduction studies, two had mild length-dependent sensory axonal neuropathy (2/8; 25%) and six had mild mixed sensorimotor axonal neuropathy (6/8; 75%). Mild distal muscle weakness and/or atrophy of the lower extremities was observed in three of six patients with sensorimotor neuropathy. Alternative causes of neuropathy were not identified. The polyneuropathy was limited to the lower extremities in five patients and was generalised in three patients. None had electrodiagnostic evidence of sensory neuronopathy, a hallmark of *RFC1*-related disease.^11^ Otherwise unexplained urinary urgency was present in 57% of patients, suggesting that autonomic dysfunction might be a feature of GAA-*FGF14*-related disease. Walking aids were used by 50% of patients (8/16) after an average disease duration of 10.8 years, whereas use of a wheelchair was rare and occurred after long-standing disease (~20 years) in two patients (2/16; 12%). Treatment with 4-aminopyridine resulted in objective and/or subjective improvement in ataxia in four of five (80%) patients.

Table 1 presents the baseline characteristics of the GAA-*FGF14*-positive and GAA-*FGF14*-negative cohorts. Comparison of all clinical features in the two cohorts revealed significantly less frequent permanent cerebellar dysarthria (2/17; 12% vs 14/26; 54%; Fisher’s exact test p=0.009) and non-significantly more frequent episodic symptoms (10/17; 59% vs 7/26; 27%; Fisher’s exact test p=0.06) in GAA-*FGF14*-positive compared with GAA-*FGF14*-negative patients. Family history of ataxia, which was positive in 59% of GAA-*FGF14*-positive patients, was significantly more frequent in GAA-*FGF14*-positive compared with GAA-*FGF14*-negative patients (59% vs 15%; Fisher’s exact test, p=0.007).

**Table 1:** Characteristics of the GAA-*FGF14*-positive and GAA-*FGF14*-negative patients

	GAA-*FGF14*-positive (n=17)*	GAA-*FGF14*-negative (n=28)†
Male sex—no. (%)	13 (76)	13 (46)
Triplet repeat count of the larger allele	343 (258–637)	62 (8–247)
Age at disease onset—years	63 (28–78)	60 (15–80)
Age at onset of gait ataxia—years	63 (37–78)	61 (30–80)
Disease duration—years	14 (4–24)	8 (2–56)
Age at last examination—years	77 (44–86)	77 (49–91)
Positive family history—no./total no. (%)	10/17 (59)	4/26 (15)
Presenting symptoms at disease onset—no. (%)‡		
Gait unsteadiness	14 (82)	26 (93)
Vertigo or dizziness	7 (41)	5 (18)
Visual disturbances (diplopia, oscillopsia, blurring)	3 (18)	1 (4)
Episodic dysarthria	3 (18)	0 (0)
Sensory symptoms	0 (0)	2 (7)
Phenotypic classification—no. (%)		
Cerebellar ataxia plus polyneuropathy	5 (29)	8 (29)
Cerebellar ataxia plus bilateral vestibulopathy	9 (52)	12 (43)
Cerebellar ataxia plus polyneuropathy and bilateral vestibulopathy	3 (18)	8 (29)
Ancillary tests—no. (%)		
Brain MRI	13 (76)	24 (86)
Nerve conduction studies	15 (88)	21 (75)
Video head impulse test	8 (47)	13 (46)

Unless specified, data are reported as median (range).

*Data on vestibular system function were missing for one patient.

†Data on vestibular system function were missing for five patients.

‡Patients may present with multiple symptoms at disease onset.

*FGF14*, fibroblast growth factor 14 gene.

## Discussion

Our study demonstrates that *FGF14* GAA repeat expansions are common in patients negative for biallelic *RFC1* repeat expansions presenting with a combination of cerebellar ataxia plus polyneuropathy and/or BVP. Compared with European cohorts of late-onset ataxia in which the frequency of GAA-*FGF14* ataxia is 10–18%,^1 2^ the frequency of 38% observed in this cohort suggests that *FGF14* repeat expansions are enriched in patients partially fulfilling criteria for CANVAS. These results may suggest a combined vulnerability of the cerebellar, peripheral nerve and vestibular systems in GAA*-FGF14*-related disease. Our study thus confirms and extends previous findings showing that BVP is part of the phenotypic spectrum of GAA*-FGF14*-related disease.^1 2^ Our estimate of the frequency of BVP in GAA-*FGF14*-related disease may even represent an underestimate, as only a relatively small proportion of patients underwent vHIT. Moreover, given the inclusion criteria of our study, the true prevalence of BVP in unselected cohorts of GAA-*FGF14*-positive patients fully assessed with vHIT remains to be established. Although the prevalence of vestibular impairment in spinocerebellar ataxias has not been well studied, this feature is not specific to GAA-*FGF14*-related disease, as it is found with variable frequency in other inherited ataxias such as *RFC1*-related disease (87–90%),^5 6^ Friedreich ataxia (53–55%)^12 13^ and spinocerebellar ataxia 3 (57–100%).^14–16^

Despite phenotypic overlap between *RFC1*-related disease and GAA-*FGF14*-related disease, certain features may help differentiate these disorders. Chronic cough, a prevalent feature in *RFC1*-related disease,^5 6^ was uncommon in our cohort. While motor neuropathy is typically absent or minimal in *RFC1*-positive patients,^5 11 17^ it co-occurred with sensory neuropathy in six of eight GAA-*FGF14* patients. Our findings also suggest that episodic symptoms—which were common in previously reported cohorts^1^—are a frequent feature in GAA-*FGF14*-positive patients, which may help to discriminate these patients from *RFC1*-positive patients in whom episodic symptoms are rare. Finally, the pattern of inheritance, which is autosomal dominant in GAA-*FGF14*-related disease and autosomal recessive in *RFC1*-related disease, may help differentiating both disorders, although acknowledging that in comparison with other dominant spinocerebellar ataxias^18^ a substantial proportion of patients with GAA-*FGF14*-related disease present sporadically (15–50%, depending on cohorts)^1^ or with seemingly recessive inheritance.

Limitations of this study include its small cohort size and the fact that only 29% (13/45) of patients underwent brain MRI, nerve conduction studies and vHIT. Since bedside head impulse test has a sensitivity of less than 70% for detecting vestibulopathy compared with vHIT,^19^ a systematic assessment of the vestibular function in phenotypically unselected GAA-*FGF14*-positive cohorts using vHIT will be necessary to fully define the frequency of vestibular hypofunction in GAA-*FGF14*-related disease in future studies. Larger natural history studies are needed to fully define the phenotypic spectrum of GAA-*FGF14*-related disease (for first in-depth phenotype and progression study, see Wilke *et al* 20) and to assess its frequency in patients meeting the proposed diagnostic criteria for clinically definite CANVAS negative for biallelic *RFC1* repeat expansions. Such studies will also be critical to evaluate the degree to which polyneuropathy is pathologically related to GAA-*FGF14*-related disease—a late-onset disorder—rather than an age-related process, given its high prevalence in the general elderly population.^21^

In conclusion, we showed that *FGF14* GAA repeat expansions are a common cause of cerebellar ataxia plus polyneuropathy and/or BVP in patients negative for biallelic *RFC1* repeat expansions, thus expanding the phenotypic spectrum of this recently described disorder. Our results further suggest that GAA-*FGF14*-related disease should be included in the differential diagnosis of *RFC1* CANVAS and disease spectrum.
